# Niches in cancer: from primary tumors to metastatic disease

**DOI:** 10.1186/s13045-026-01858-8

**Published:** 2026-10-16

**Authors:** Zheng Chao, Xiaojing Wang, Zhihua Wang, Kongming Wu, Le Li

**Affiliations:** 1 1Department of Urology, Hubei Provincial Clinical Medical Research Center for Minimally Invasive Treatment of Urology, Tongji Hospital, Tongji Medical College, State Key Laboratory for Diagnosis and Treatment of Severe Zoonotic Infectious Diseases, Huazhong University of Science and Technology, Wuhan, 430030 China; 2 2Department of Infectious Diseases, Tongji Hospital, Tongji Medical College, State Key Laboratory for Diagnosis and Treatment of Severe Zoonotic Infectious Diseases, Huazhong University of Science and Technology, Wuhan, 430030 China; 3 3Taikang Tongji (Wuhan) Hospital, Wuhan, 430000 China; 4 4Department of Oncology, Tongji Hospital of Tongji Medical College, Huazhong University of Science and Technology, Wuhan, 430030 China

**Keywords:** Cancer niche, Tumor microenvironment, Pre-metastatic niche, Metastatic niche, Therapy resistance, Cancer mechanobiology

## Abstract

Cancer progression does not occur in isolation: malignant-cell states and fates are shaped by specialized microenvironments, or cancer niches. Within primary tumors, vascular, stromal, immune and neural interactions create local hypoxic, metabolic and mechanical conditions that together influence malignant-cell behavior. These local environments sustain stem-like states, regulate immune surveillance and vascular remodeling, and influence dissemination and treatment response. Tumor-derived extracellular vesicles and other systemic signals connect primary tumors to distant organs, conditioning tissues before tumor-cell arrival and subsequently modulating disseminated tumor-cell seeding, dormancy, reactivation and outgrowth. Cancer niches therefore operate across spatial scales and disease stages, shaping tumor behavior in ways that cannot be explained by tumor-cell-intrinsic properties alone. Advances in single-cell and spatial profiling, organoid systems and intravital imaging are revealing their cellular composition, local architecture and evolution. In this review, we integrate primary, pre-metastatic and metastatic niche biology, examine how local environments evolve and influence one another during cancer progression, compare organ-specific metastatic niches and assess emerging therapeutic strategies. Effective niche-directed therapy will require identifying the interactions that sustain disease, showing that treatment disrupts them and determining which patients are most likely to benefit. Greater spatial, temporal and functional resolution may reveal new therapeutic vulnerabilities and create opportunities to prevent relapse and achieve more durable disease control.

## Introduction

More than a century ago, Paget’s “seed and soil” hypothesis proposed that metastatic growth depends on the compatibility between disseminated tumor cells and the tissues in which they arrive [1]. This idea anticipated the modern recognition that cancer behavior is determined not only by malignant-cell-intrinsic properties but also by the surrounding tumor microenvironment (TME). The TME encompasses nonmalignant cells, extracellular matrix (ECM), soluble factors, metabolites and physical conditions, and has become indispensable to understanding tumor development and treatment response. Yet its breadth creates an important limitation: an inventory of what is present within and around a tumor does not reveal which components form functional relationships or which interactions alter malignant-cell behavior. The stem-cell niche offers a more focused model of such organization. In the bone marrow, spatially arranged osteoblastic and stromal elements regulate stem-cell quiescence, self-renewal and differentiation [2, 3]. Tumors establish or co-opt comparable relationships to support local growth, adaptation to treatment and metastatic colonization [4–6]. Thus, whereas the TME describes the broader cellular, molecular and physical milieu, a cancer niche comprises the spatially and functionally connected elements that maintain or alter a particular malignant-cell state or fate.

In tumors, such functional organization often takes shape around recurring cellular and tissue structures. Blood vessels are not simply conduits for oxygen and nutrients: endothelial cells, pericytes and the perivascular matrix also regulate tumor-cell trafficking, stemness, survival and dormancy [7, 8]. Fibroblasts and the ECM provide an additional scaffold, shaping tissue architecture and transmitting biochemical and mechanical signals among vascular, immune and malignant cells [9, 10]. Vessel state and stromal organization together influence immune-cell entry, whereas interactions among malignant cells, antigen-presenting cells, lymphocytes and myeloid populations determine whether antitumor responses are initiated, excluded or suppressed [11–13]. Neural inputs further modify vascular, stromal and immune behavior through neurotransmitters, neuropeptides and reciprocal signaling between nerves, tumor cells and other local populations [14, 15]. These niches are named for the cells or structures that organize local interactions, rather than for the components that are simply most abundant.

Within these structures, tumor growth progressively alters the conditions under which cells interact. An imbalance between oxygen consumption and vascular delivery creates hypoxia and steep oxygen gradients. Competition and metabolic exchange among malignant, immune and stromal cells also reshape the local availability of glucose, lipids and amino acids and promote metabolite accumulation [16, 17]. In parallel, proliferative expansion, matrix deposition and cross-linking increase tissue stiffness and solid stress; compression of blood and lymphatic vessels alters interstitial fluid pressure and flow, further limiting perfusion, nutrient delivery and immune-cell access. These overlapping conditions generate hypoxic, metabolic and mechanical niches. Cells respond through partially overlapping networks, including hypoxia-inducible factor (HIF) signaling during oxygen limitation, AMPK–mTOR signaling in response to energy and nutrient availability, and YAP/TAZ signaling in response to mechanical inputs.

For malignant cells, the consequences of these changing conditions are expressed as shifts in phenotype and fate. Cancer stem cell (CSC) niches maintain or regenerate tumor-propagating, stem-like states with self-renewal capacity and increased tolerance of stress [18]. These states may pre-exist, be selected under microenvironmental or therapeutic pressure, or arise through reversible plasticity; in turn, stem-like malignant cells can remodel the niche that supports them [19, 20]. The accumulation of VEGFR1-positive bone-marrow-derived progenitors at future metastatic sites provided early experimental evidence that distant tissues can be conditioned before tumor-cell arrival [21]. Subsequent studies have shown that tumor-derived extracellular vesicles (EVs), cytokines and metabolites cooperate with mobilized host cells to recruit inflammatory and immunosuppressive populations and remodel vascular, lymphatic and extracellular-matrix compartments in organ-specific ways [4, 22–24]. These factors collectively promote pre-metastatic niche formation but do not constitute independent niche categories. After arrival, evolving metastatic niches shape whether disseminated tumor cells are eliminated, persist in dormancy or eventually reactivate and form overt metastases [25, 26].

**Fig. 1:**
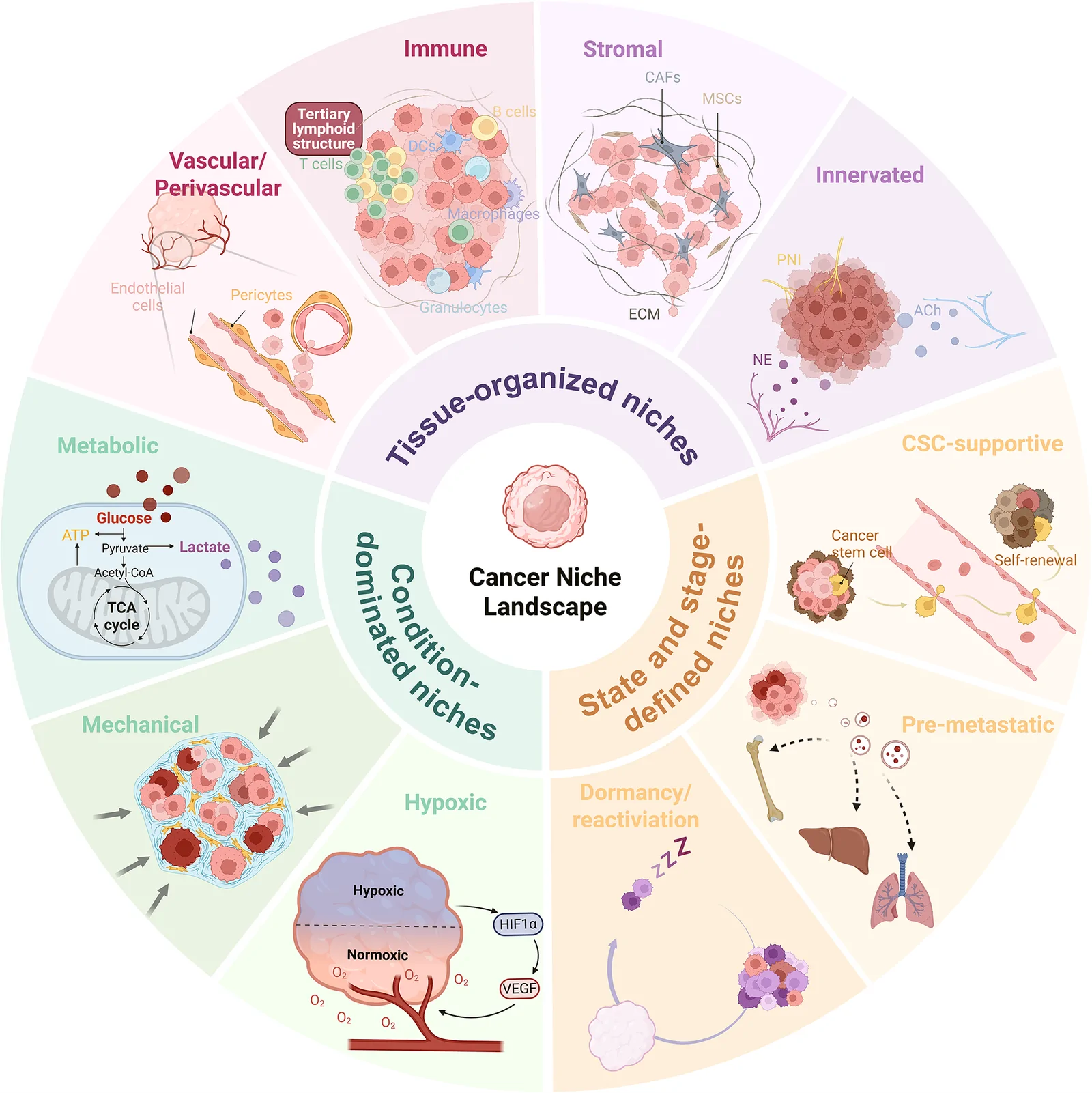
The cancer niche landscape. Cancer niches can be described by the tissue structures that organize local interactions, the physiological and biophysical conditions that emerge within them, and the malignant-cell states or stages of disease that they support. Extracellular vesicles and other systemic signals connect niches across space and time. ACh, acetylcholine; ATP, adenosine triphosphate; CAF, cancer-associated fibroblast; CSC, cancer stem cell; DC, dendritic cell; ECM, extracellular matrix; EV, extracellular vesicle; HIF-1α, hypoxia-inducible factor 1α; MSC, mesenchymal stromal cell; NE, norepinephrine; PNI, perineural invasion; TCA, tricarboxylic acid; VEGF, vascular endothelial growth factor

Niches are neither spatially separate nor mutually exclusive (Fig. 1). A perivascular region may also be hypoxic, nutrient restricted and mechanically compressed while supporting stemness or dormancy; an immune niche is likewise shaped by vascular access, stromal barriers and metabolic competition. Individual tumor regions can therefore combine several niche features, and these relationships change as malignant cells adapt, disseminate and colonize distant organs. Such overlap helps explain why similar malignant phenotypes can arise in different tissues and at different stages of disease [27, 28].

In this review, we follow cancer niches from primary tumors through pre-metastatic conditioning, metastatic colonization, dormancy and reactivation. We first examine the vascular, stromal, immune and neural interactions that organize local tumor environments, and the hypoxic, metabolic and mechanical conditions that emerge within them. We then compare metastatic niches across major organs and consider malignant bone-marrow niches as a non-metastatic counterpart. We also discuss how emerging technologies can resolve and test these interactions before examining niche-mediated therapy resistance and propose the future for clinical translation (Table 1).

**Table 1:** Conceptual organization and defining features of cancer niches

Organizing dimension	Niche designation	Defining basis	Representative organizers, conditions and signaling	Principal biological roles
Tissue-organized niches	**Vascular/perivascular niche**	Spatial interfaces formed by blood vessels and adjacent cells	Endothelial cells, pericytes and perivascular matrix; VEGF, Notch and CXCL12-CXCR4 signaling	Perfusion and tumor-cell trafficking; immune-cell entry; stem-like states; dissemination and dormancy
	**Stromal niche**	Fibroblast- and ECM-organized tissue scaffold	CAFs, MSCs and ECM; TGF-β, FAP, LOX and FAK-Src signaling	Tissue architecture; tumor growth and invasion; immune exclusion; treatment response
	**Immune niche**	Spatially organized interactions between malignant and immune cells	T cells, B cells, antigen-presenting cells, TAMs, MDSCs, Tregs and TLS; cytokines and immune checkpoints	Immune surveillance, exclusion and suppression; tumor-cell plasticity; response to immunotherapy
	**Innervated niche**	Local circuits organized by neural fibers and reciprocal tumor-neural signaling	Autonomic and sensory nerves; neurotransmitters, neuropeptides, ADRB2, cholinergic signaling and NGF-TrkA	Tumor growth and invasion; vascular, stromal and immune regulation; perineural invasion
Condition-dominated niches	**Hypoxic niche**	Low oxygen and steep oxygen gradients created by an imbalance between consumption and vascular delivery	HIF-1α and HIF-2α, VEGF and redox-regulatory pathways	Metabolic adaptation; angiogenesis; plasticity; immune evasion and treatment resistance
	**Metabolic niche**	Local nutrient availability, metabolite exchange and redox state	Competition for glucose, lipids and amino acids; lactate and adenosine; AMPK-mTOR signaling	Tumor and stromal adaptation; immune dysfunction; organ colonization and treatment resistance
	**Mechanical niche**	Local forces and material properties generated by tumor growth and tissue remodeling	ECM stiffness and viscoelasticity, solid stress, compression, interstitial fluid pressure and flow; integrin-FAK, Rho-actomyosin and YAP/TAZ signaling	Tumor-cell plasticity and invasion; vascular compression; impaired immune access and drug delivery
State- and stage-defined niches	**CSC-supportive niche**	Interactions that maintain or regenerate stem-like malignant-cell states	Endothelial cells, pericytes, CAFs, macrophages and ECM; Notch, WNT, BMP/TGF-β, Hedgehog and YAP/TAZ signaling	Self-renewal and tumor propagation; plasticity; treatment tolerance and relapse
	**Pre-metastatic niche**	Conditioning of distant tissues before tumor-cell arrival	Primary-tumor-derived EVs, cytokines and metabolites; mobilized host cells; vascular, immune, stromal and ECM remodeling	Tumor-cell retention, extravasation and early survival; organ-selective colonization
	**Dormancy/reactivation niche**	Local signals that maintain DTC quiescence or permit renewed growth	Perivascular interfaces, ECM and immune surveillance; BMP, TGF-β and p38-ERK signaling; inflammation and angiogenic remodeling	DTC persistence; dormancy; reactivation and late relapse
	**Metastatic niche**	Organ-specific interactions after tumor-cell arrival	Resident vascular, stromal, immune and metabolic programs; reciprocal tumor-host remodeling	Early adaptation and survival; colonization and outgrowth; therapy response

ADRB2, β2-adrenergic receptor; AMPK, AMP-activated protein kinase; BMP, bone morphogenetic protein; CAF, cancer-associated fibroblast; CSC, cancer stem cell; DTC, disseminated tumor cell; ECM, extracellular matrix; EV, extracellular vesicle; FAK, focal adhesion kinase; FAP, fibroblast activation protein; HIF, hypoxia-inducible factor; LOX, lysyl oxidase; MDSC, myeloid-derived suppressor cell; MSC, mesenchymal stromal cell; NGF, nerve growth factor; TAM, tumor-associated macrophage; TGF, transforming growth factor; TLS, tertiary lymphoid structure; Treg, regulatory T cell; VEGF, vascular endothelial growth factor

## Cellular and structural organization of cancer niches

Cancer niches often develop around tissue structures that coordinate exchange, support and surveillance. Tumors reshape vascular, stromal, immune and neural relationships to control access to nutrients, signals and neighboring cells. These systems are interdependent: vessels regulate perfusion and leukocyte entry; stromal cells and extracellular matrix position vascular and immune cells; immune populations remodel vessels and matrix; and neural signals modulate each process. A single region may combine several forms of tissue organization.

### Vascular and perivascular niches

Vascular remodeling shapes nutrient and oxygen delivery, tumor-cell entry into the circulation, immune-cell trafficking and drug exposure. Primary tumors secure a blood supply through sprouting angiogenesis and vessel co-option; some experimental models also implicate vasculogenic mimicry and endothelial-like tumor-cell transdifferentiation [16, 29]. Placental growth factor-positive endothelial tip cells with mesenchymal features are enriched in immune-depleted microenvironments and associated with poor prognosis [30]. Endothelial cells are also instructive: Notch, WNT and bone morphogenetic protein (BMP) angiocrine signals can alter tumor-cell fate independently of bulk perfusion [7].

Vascular phenotype can matter as much as vessel density. Disorganized, poorly covered and hyperpermeable vessels can facilitate dissemination while producing uneven perfusion that limits drug delivery and lymphocyte entry. By contrast, partial restoration of endothelial junctions, pericyte coverage and flow can create a transient normalization window [31]. This distinction helps explain why anti-angiogenic treatment can either improve access for chemotherapy and immune cells or deepen hypoxia when vascular pruning is excessive [32]. Dose, timing and tumor context determine which effect predominates; clinical benefit from combined vascular and immune blockade does not by itself prove that normalization occurred in an individual patient [29, 33, 34].

Organ architecture constrains these vascular functions. In the brain, endothelial cells, pericytes, astrocytes, and microglia form a neurovascular unit whose tight junctions and inflammatory responses regulate extravasation and immune access [35]. Brain metastases can exploit both sprouting vessels and pre-existing vasculature, illustrating how recipient-organ structure limits the vascular strategies available to tumor cells.

The perivascular niche integrates stemness, immune regulation, and dormancy. Endothelial Notch, Wingless-related Integration Site (WNT) and BMP signals support CSC self-renewal [19], and an arteriolar LPA–PKD1 circuit provides an additional stemness cue [36], whereas stem-like pericyte-lineage cells can recruit suppressive myeloid cells [37]. Vascular state also switches disseminated tumor cell (DTC) fate: stable microvessels provide thrombospondin-1-associated dormancy signals, while sprouting endothelial tips release TGF-β1 and periostin to promote outgrowth [38]. In bone, vascular and endosteal interfaces provide attachment and shelter [39, 40]. Across tissues, the perivascular niche recurs as a functional interface with a variable cellular composition.

Organ context further determines endothelial instruction. In lung, endothelial signals can shift disseminated cells between intravascular growth and latency [41]. In liver, continuous IFNα sensing by sinusoidal endothelium contributes to an antimetastatic barrier [42]. These findings show that endothelium can promote, restrain or delay colonization. Anti-angiogenic and endothelial-directed strategies may perturb these functions [43], but bulk vascular markers cannot identify which role dominates in a given lesion.

### Stromal niches

Stromal niches are organized largely through fibroblast–matrix interactions, but neither fibroblasts nor the ECM exerts uniform effects across tumors. Cancer-associated fibroblasts (CAFs) remodel ECM, provide metabolic support, promote epithelial–mesenchymal transition (EMT) and regulate antitumor immunity [9]. Distinct CAF populations can exert opposing effects on tumor progression and treatment response, making selective targeting more plausible than indiscriminate fibroblast depletion [44].

In primary tumors, CAFs create physical and biochemical barriers through ECM deposition, matrix stiffening, vascular compression and paracrine signaling [45, 46]. CAF-derived EV cargo provides another route to resistance: miRNAs, lncRNAs and circRNAs can alter PI3K–AKT and WNT–β-catenin signaling, drug efflux and DNA repair [47]. These mechanisms have been reported across chemotherapy, targeted therapy, radiotherapy and immunotherapy, and CAF phenotypes can change under treatment pressure [47, 48]. Their effects consequently depend on phenotype, location and secreted cargo rather than fibroblast abundance alone.

At metastatic sites, organ-resident fibroblasts execute distinct stromal programs. In lung, COX-2-expressing adventitial fibroblasts generate a prostaglandin E2 (PGE2)-dependent immunosuppressive state that is reinforced by tumor-derived interleukin-1β (IL-1β); genetic *Ptgs2* deletion or combined EP2 and EP4 inhibition reverses this phenotype in preclinical models [49]. Metastasis-associated lung fibroblasts also produce CXCL9 and CXCL10 in response to tumor-derived IL-1 [50]. In liver, activated stellate cells deposit ECM and recruit immunosuppressive cells [51], whereas TSPAN4-positive fibroblasts can coordinate niche assembly through migrasome-mediated communication [52]. Tissue, phenotype and spatial context—not fibroblast lineage alone—determine stromal function.

A pan-cancer single-cell and spatial atlas defined 39 non-immune stromal states linked to distinct functions, locations and clinical features and connected progenitor states with pericyte diversity and vascular support [30]. These data expose a therapeutic paradox: broad fibroblast depletion may remove tumor-restraining and homeostatic functions. Spatially restricted CAF programs or signaling circuits may therefore be more tractable targets, while their effects on vessel integrity and leukocyte access help establish local immune architecture.

### Immune niches

The composition and spatial organization of immune cells influence tumor progression and treatment response [53]. Tumors are commonly described as inflamed, excluded or immune-desert [53, 54], but these patterns are not yet validated treatment-selection assays. Deficient priming and trafficking contribute to T-cell desertification and exclusion, whereas restored antigen presentation, leukocyte infiltration and greater tertiary lymphoid structure (TLS) abundance accompany response [55]. Even among mismatch repair (MMR)-deficient tumors, immune-desert phenotypes are associated with poor responsiveness to checkpoint inhibition [56], showing that immune architecture must be interpreted together with tumor genotype and treatment context.

TLSs provide one example of how immune organization shapes local function. These aggregates support lymphocyte recruitment, activation and differentiation [57, 58], and their presence and maturation have been associated with prognosis and response to immune-checkpoint blockade [59]. TLSs can sustain local antitumor immunity in some tumors with low mutational burden [60], whereas pan-cancer analyses link high TLS scores to higher mutational and neoantigen burden [61]. TLS abundance has also been associated with longer survival [62] and higher response rates after checkpoint inhibition [55]. These findings support the development of TLS-based spatial biomarkers, but also show why standardized assessment of location, maturity and density is needed [63].

TLS organization can also change during treatment [64], raising the possibility that it contributes to response rather than merely marking a pre-existing immune phenotype. More mature or denser TLSs have been associated with favorable outcomes in head and neck squamous cell carcinoma [65] and colorectal cancer [66], although these relationships vary across tumor types [64]. Toll-like receptor agonists [67] and engineered exosomes [68] can induce TLS-like organization experimentally. In non-small-cell lung cancer (NSCLC) brain metastases, combined checkpoint blockade was associated with intracranial control, while mouse studies showed CD8-positive T-cell expansion and TLS-like structures near meningeal and perivascular spaces [69]. The therapeutic value and appropriate clinical setting for deliberate TLS induction nevertheless remain uncertain [70].

Systemic tumor-derived signals can reshape immune organization in distant tissues before tumor-cell arrival, reducing natural killer (NK)-cell activity and expanding regulatory or suppressive myeloid populations [4, 6, 71]. Extracellular vesicles and soluble factors connect altered bone-marrow output with responses in recipient tissues [4]. In experimental lung models, epithelial TLR3 activation [72], granulocyte colony-stimulating factor (G-CSF)-biased myelopoiesis [4] and complement-dependent fibroblast–myeloid crosstalk [4] show how circulating signals are interpreted by local cells [73] .

Recipient organs interpret systemic conditioning differently. Liver metastases are often relatively tolerogenic and myeloid-rich, whereas lung metastases can retain a more inflammatory environment [74]. Treatment response also depends on the surrounding vascular and stromal architecture [75]. The immune state of a metastasis is inseparable from the anatomy and resident-cell composition of its host organ.

### Innervated niches

Neural regulation of cancer extends beyond the presence of nerves within a tumor [14, 76–78]. Tumors recruit nerves through axonogenesis and perineural invasion [79], while autonomic and sensory fibers alter malignant, stromal and immune behavior [80]. Tumor-derived trophic and guidance cues can promote neural recruitment [80] and establish reciprocal tumor–nerve circuits [14] whose effects vary by nerve type and organ [81].

The outcome of innervation depends on the transmitter–receptor pair, tumor lineage and the local cells that relay the signal [82, 83]. Nociceptor-derived CGRP can promote CD8-positive T-cell exhaustion in melanoma [84], whereas substance P activates a TACR1–TLR7 circuit that favors invasion in breast cancer [85]. Innervation at the invasive front of prostate cancer [86] and neurotrophic signaling in pancreatic cancer [87] further demonstrate this tissue specificity. Neural effects should be interpreted in the context of the relevant signaling circuit and receptor dependency.

**Fig. 2:**
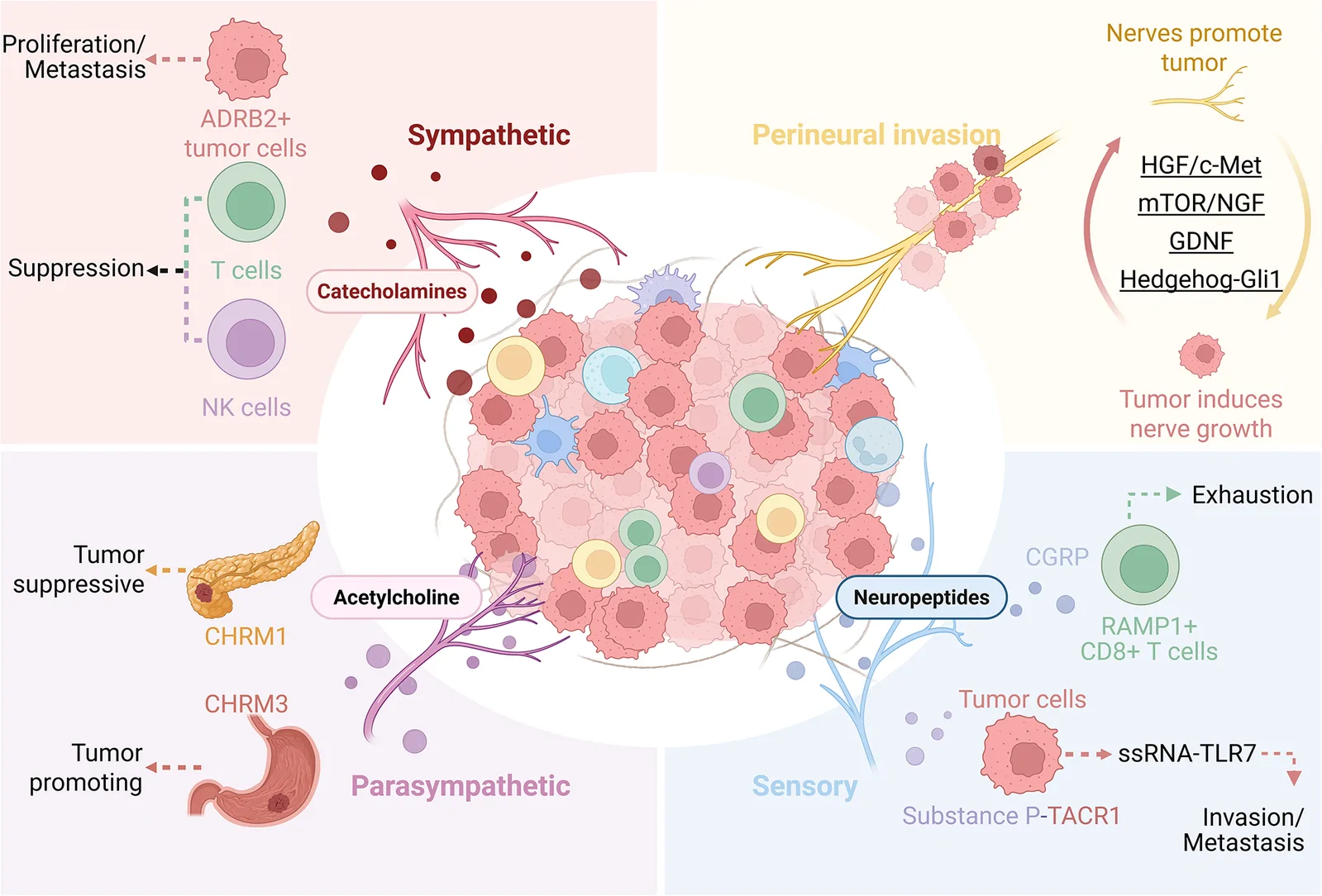
Neural regulation of cancer niches. Neural signals regulate tumor, immune and stromal compartments, while tumors reciprocally remodel and exploit peripheral nerves. Sympathetic catecholamine signaling can promote tumor progression and suppress T-cell and NK-cell activity. Parasympathetic acetylcholine signaling is context dependent, with CHRM1- and CHRM3-associated pathways exerting distinct effects across cancer types. Sensory neuropeptides can directly promote invasion or modulate antitumor immunity, including through CGRP–RAMP1 signaling in CD8-positive T cells. Reciprocal tumor–nerve signaling involving HGF–c-MET, mTOR, NGF, GDNF and Hedgehog–GLI1 pathways promotes nerve growth, neural remodeling and perineural invasion. These effects depend on tumor type, tissue context, receptor expression and disease stage. CGRP, calcitonin gene-related peptide; GDNF, glial cell line-derived neurotrophic factor; HGF, hepatocyte growth factor; NGF, nerve growth factor; NK, natural killer; RAMP1, receptor activity-modifying protein 1

Sympathetic catecholamines activate ADRB2–cAMP–PKA signaling in malignant and immune cells. Chronic stress promotes gastric-cancer progression in preclinical models [88], whereas propranolol suppresses colorectal-tumor growth and increases CD8-positive T-cell activity [89]. β-adrenergic blockade can also enhance anti-cytotoxic T-lymphocyte-associated protein 4 (CTLA-4) responses by reducing myeloid-derived suppressor cells (MDSCs) [90, 91] and redirect CD4-positive T cells toward cytotoxicity [92] (Fig. 2). In these experimental settings, immune modulation contributes substantially to the tumor-promoting effects of sympathetic signaling.

Parasympathetic cholinergic signaling is likewise context dependent. CHRM1 signaling suppresses tumorigenesis and stemness in pancreatic-cancer models [93], whereas vagal CHRM3 signaling promotes gastric-cancer growth [94]. Neural crosstalk also contributes to cancer pain [95] and can suppress NK- and T-cell function [96], while tumor-derived trophic cues recruit further axons [97]. The direction of the response varies with receptor, tissue and tumor type, arguing against uniform neuromodulation.

Perineural invasion (PNI)—the spread of cancer along nerves—is associated with aggressive behavior, treatment resistance and poor outcomes [98] and provides a route of dissemination distinct from vascular or lymphatic spread [95]. Tumor-driven neoaxonogenesis [80] and macrophage-to-neuron-like transitions [98] expand the range of cell types involved in this process. Mechanistically defined circuits include HGF–c-MET–mTOR–NGF signaling [99], exosomal lncRNA *XIST* and LRP1-dependent regulation of GDNF in pancreatic cancer [100], and Hedgehog–GLI1 or exosomal circRNA signaling [101].

β-adrenergic blockade [80], disruption of NGF–TRK signaling [96] and modulation of treatment-induced nociceptive pathways [79] provide possible routes to intervention. Extracellular vesicles can also recruit and reprogram axons [14], linking local innervation to communication between tissues. The field nevertheless remains dominated by preclinical evidence, and the relevant circuits vary by tumor, organ and receptor. Biomarkers of functional innervation will be needed before neuromodulation can be used rationally.

## Physiological and biophysical states within cancer niches

The structures described above also shape the physiological and biophysical conditions under which cells interact. Abnormal perfusion and high cellular demand generate oxygen and nutrient gradients, metabolic exchange alters redox balance and local metabolite availability, and matrix remodeling and tumor expansion change tissue stiffness, pressure and fluid flow. These conditions rarely occur in isolation: hypoxia, metabolic stress and mechanical stress often coexist within the same vascular, stromal or immune environment and reinforce one another. Each state is nevertheless associated with a characteristic molecular response, and the balance of pathway activity can help indicate which condition predominates locally, even though no single pathway is exclusive to one state.

### Hypoxic niches

A hypoxic niche forms when oxygen consumption exceeds vascular delivery, creating regions of sustained oxygen limitation and steep oxygen gradients. Malignant and nonmalignant cells adapt through HIF-dependent transcriptional programs that regulate metabolism, angiogenesis, immune evasion and treatment response [16, 17]. HIF-1α and HIF-2α have overlapping but context-dependent functions [102, 103]: HIF-1α often predominates during acute oxygen deprivation, whereas HIF-2α can support longer-term adaptation, vascular remodeling and expression of stemness-associated factors such as OCT4, SOX2 and NANOG [16, 104]. Their relative importance varies with tumor type and disease stage and cannot be reduced to a universal linear “HIF switch” [105, 106].

HIF programs connect oxygen sensing to the wider niche by coordinating glycolysis, oxidative phosphorylation and lipid metabolism with angiogenic and secretory signals that remodel stromal and immune compartments [6, 17, 107]. These responses can support treatment resistance, although the relevant targets and biomarkers remain cancer- and context-specific [107–109]. Clear-cell renal cell carcinoma provides a clinically defined example: von Hippel–Lindau loss constitutively activates HIF-2α, and selective HIF-2α inhibition has therapeutic activity [17, 110]. HIF-2α can also promote metastatic signaling through MCJ-dependent prolegumain secretion and MMP2 activation [111]. This example establishes a genotype-linked HIF-2α dependency, not a general indication for HIF inhibition in all hypoxic tumors.

Hypoxia also changes neighboring immune and stromal cells. HIF-dependent programs can impair T- and natural-killer-cell function, expand suppressive populations and promote angiogenesis [17, 107, 110]. Disorganized vessels then sustain oxygen limitation, while acidity and metabolic products further restrict immunity. Hypoxia-associated signals can also extend beyond the local tissue: patient-derived vesicles can induce HIF-2α accumulation in monocytes [112], and hypoxia-regulated secretomes can condition distant organs [29]. A local oxygen state can have metabolic, immune and systemic consequences.

### Metabolic niches

Metabolic niches arise when nutrient supply, cellular demand and exchange create local patterns of resource availability, waste accumulation and redox stress [113]. Primary tumors can modify these conditions at distant sites: breast-cancer-derived miR-122 suppresses glucose uptake in lung and brain cells before colonization [114]. Tumor-derived vesicles can also induce glycolytic, PD-L1-positive macrophages through TLR2–MyD88–NF-κB signaling [115]; corresponding associations between macrophage PD-L1, tumor GLUT1 and YKT6 have been observed in histologically uninvolved lymph nodes [115].

A metabolic niche cannot be inferred from a single nutrient. In a comprehensive mouse-tissue survey, quantification of 124 metabolites showed that no metabolite alone explained patterns of metastatic colonization [116]. Colonization instead reflected the interaction between tissue nutrients and tumor-intrinsic programs, with purine synthesis recurring across several tissues [116]. Hypoxia-driven glycolytic and lipid programs further link metabolic adaptation to immune suppression and treatment resistance [16, 17]. Metastatic compatibility is better predicted by the interaction between tumor state and tissue metabolism than by any single metabolite.

Organ-specific dependencies show how metabolic compatibility is established. Cancer cells can obtain calcium from osteogenic cells in bone [117] and exploit local iron sources in bone [118], whereas pulmonary colonization can be influenced by HAO1-dependent oxalate metabolism [119] and an endothelial palmitic-acid-rich state [120]. The latter was selective for lung in the experimental model and could be suppressed by glucagon-like peptide-1 receptor agonism [120]. Each exchange is conditioned by the vascular, stromal and immune architecture of the recipient organ.

### Mechanical niches

Tumor growth and tissue remodeling generate matrix stiffness, viscoelasticity, solid stress, compression, interstitial fluid pressure and shear. Cells sense these forces through integrins, cytoskeletal tension and YAP/TAZ-associated pathways that regulate proliferation, survival, migration and plasticity [31]. A mechanical niche arises when these forces alter cell behavior within a defined tissue context.

Solid stress generated by tumor expansion and matrix accumulation can compress blood and lymphatic vessels, reducing perfusion and increasing interstitial fluid pressure [45, 46]. The resulting changes in oxygen and nutrient delivery, drug penetration, immune-cell access and interstitial flow couple mechanics to vascular, metabolic and immune function. Mechanical regulation is well supported experimentally, but its contribution remains difficult to quantify directly in patient tumors.

Extracellular-matrix remodeling is a major source of these physical changes [121]. Tumor and stromal cells alter matrix composition, degradation and stiffness, with consequences for invasion and immune-cell function [121]. Lysyl oxidase family members provide a well-defined example: collagen cross-linking increases matrix stiffness and fibrotic remodeling and can promote metastatic progression [122]. In colorectal cancer, matrix stiffness can also drive progression through epigenetic reprogramming, as stiffening-induced *ALKBH1* erases DNA 6 mA and disrupts *TP53*-driven *CDKN1A* transcription [123].

Mechanical remodeling also occurs in distant tissues. Pulmonary lysyl oxidase expression can promote lung seeding [124]. Activin A provides one route to pulmonary fibrosis and pre-metastatic conditioning [125]. Changes in stiffness, collagen organization and fibronectin networks can then influence organ-specific colonization and metastatic outgrowth [126]. These studies connect matrix remodeling to metastasis through measurable physical changes, although the relative contribution of mechanics and associated biochemical signals is often difficult to separate [127].

## Niche evolution during cancer progression

Cancer niches change as disease progresses from local growth to dissemination and metastatic colonization. The cellular structures and local conditions described above are not static backdrops. They are remodeled as tumors grow, encounter treatment and spread, allowing similar vascular, stromal or immune interactions to support different malignant-cell states at different stages of disease. Microenvironmental and therapeutic pressures can select or induce stem-like and treatment-tolerant states, while primary tumors condition distant tissues before dissemination [5]. Arriving cells may then be eliminated, remain dormant or adapt sufficiently to grow [83, 128]. These overlapping events can recur and do not form an invariant sequence.

**Fig. 3:**
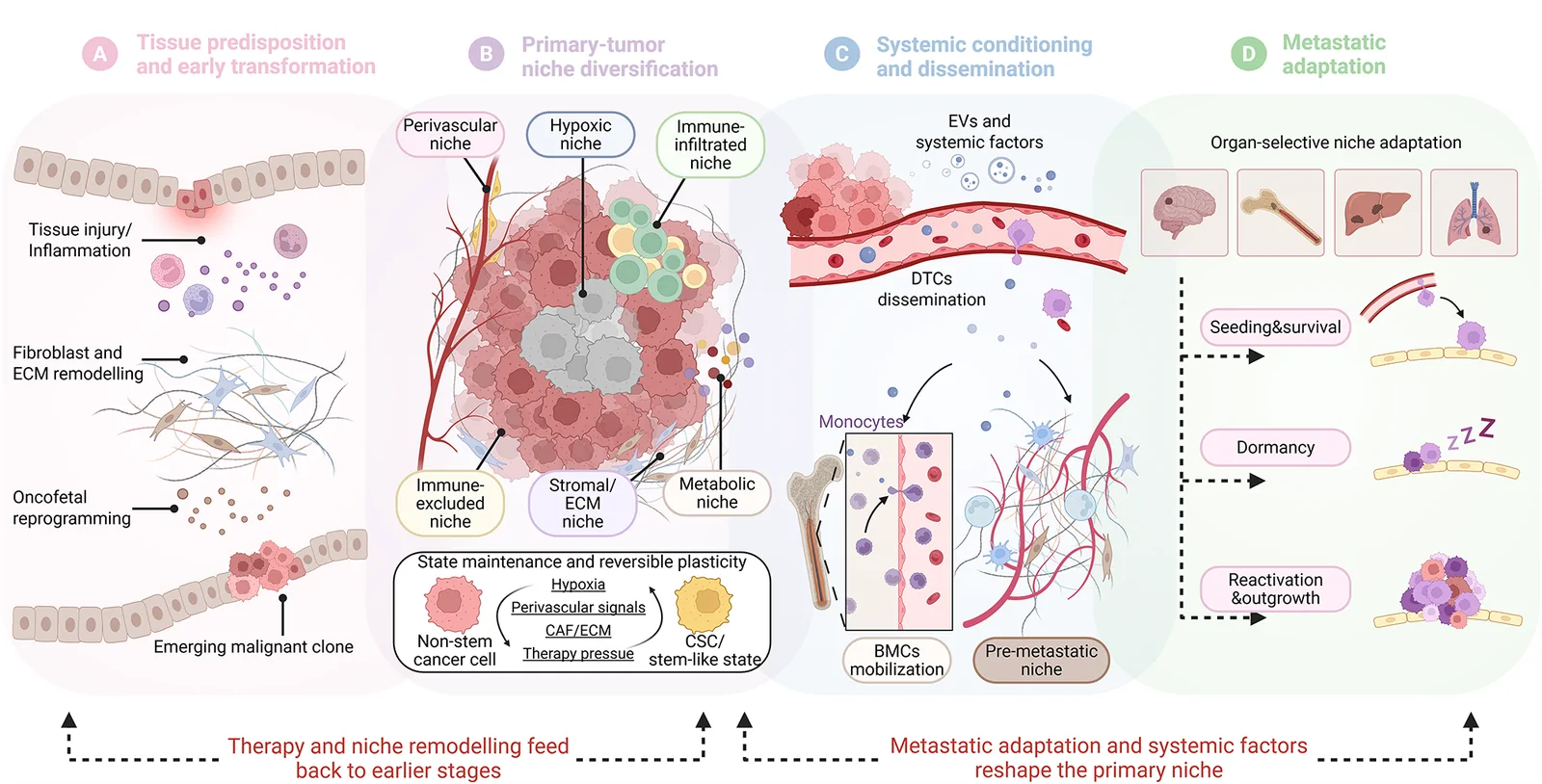
Niche evolution across the cancer trajectory. Cancer niches evolve through overlapping and context-dependent stages. **(A)** Tissue predisposition and early transformation. Injury, inflammation, fibroblast activation, ECM remodeling and oncofetal reprogramming can generate a permissive tissue state. **(B)** Primary-tumor niche diversification. Growing tumors develop heterogeneous vascular, hypoxic, immune, stromal and metabolic niches that support tumor-cell states and plasticity. **(C)** Systemic conditioning and dissemination. Tumor-derived EVs and soluble factors mobilize bone-marrow-derived cells, remodel distant tissues and promote pre-metastatic niche formation. **(D)** Metastatic adaptation. Following dissemination, DTCs interact with organ-specific niches, leading to survival, dormancy, reactivation or metastatic outgrowth. Dashed arrows indicate feedback through which therapy, systemic signals and metastatic adaptation reshape existing niches. These stages are partially reversible and do not necessarily occur in a fixed order. BMC, bone-marrow-derived cell; CAF, cancer-associated fibroblast; CSC, cancer stem cell; DTC, disseminated tumor cell; ECM, extracellular matrix; EV, extracellular vesicle

Different niche terms capture distinct points along this trajectory. They do not denote wholly separate compartments, but emphasize the particular malignant-cell state or stage of disease supported by these changing interactions. CSC-supportive niches maintain or regenerate stem-like malignant cells, whereas pre-metastatic niches arise in distant tissues before tumor-cell arrival [129]. Once disseminated cells reach a recipient organ, their interaction with the local tissue may support dormancy, reactivation or progressive growth [23, 130]. Extracellular vesicles, soluble factors, metabolites, platelets and mobilized host cells connect these sites through local and systemic communication (Fig. 3).

### Cancer stem cell-supportive niches

CSC-supportive niches maintain or regenerate malignant cells with self-renewal capacity and the ability to recreate tumor heterogeneity [131, 132]. Stromal, endothelial and ECM signals can preserve these cells, whereas reversible plasticity—including partial EMT and subsequent reversion toward epithelial growth programs during metastatic outgrowth—can generate or re-establish stem-like properties [19, 20]. CAF- and macrophage-derived cues provide one source of this plasticity [133, 134]. At the invasive front of colorectal cancer, trophocyte-like fibroblasts, a state with epithelial-supportive features, induce oncofetal transitions through direct interaction with tumor organoids [133]. The clinically relevant problem is thus not only persistence of a discrete CSC population, but also regeneration of stem-like phenotypes after treatment or during progression.

WNT signaling shows how this regenerative capacity is sustained. β-catenin-dependent transcription supports stemness and can alter interactions with the immune compartment [19]. In colorectal cancer, LGR5-positive CSCs depend on stromal WNT signals, and their ablation can cause tumor regression [135]. The response is often transient, however, because LGR5-negative cells can reacquire LGR5 expression and restore growth [135]. Many colorectal cancer metastases are likewise seeded by LGR5-negative cells that later reconstruct an epithelial hierarchy [135]. These observations place plasticity, rather than marker identity alone, at the center of the CSC-niche relationship.

CSC markers such as CD44, CD133, LGR5 and ALDH1A1 are better viewed as features of context-dependent states than as fixed identities. Reduced antigen presentation and PD-L1-associated phenotypes have been observed in marker-defined CSC populations [136]. Stem-like cells can also evade immunity through altered major histocompatibility complex (MHC) expression, suppressive cytokines, myeloid recruitment and QSOX1-dependent reactive oxygen species (ROS) production [129]. Conversely, CD8-derived IFNγ can reinforce stemness through FGF2–WNT–β-catenin signaling [129]. Marker expression, immune escape and treatment tolerance may reinforce one another, supporting strategies that target both state-specific dependencies and regenerative niche signals [137].

Perivascular signals provide one setting for stem-like phenotypes. In glioblastoma, tumor stem-like cells reinforce angiogenic and immunosuppressive vascular programs [138], including fetal-like endothelial states [138]. Related interactions occur in other solid tumors [138], and plastic tumor cells can form vessel-like channels [138]. Endothelial LPA–PKD signaling [36] and CXCL12–CXCR4-dependent retention [37] further illustrate how malignant cells use vascular interfaces to sustain plasticity.

Low-oxygen regions provide another setting in which HIF-dependent programs support quiescence, metabolic adaptation and treatment tolerance [16, 17, 139]. Because oxygen, vascular and stromal signals form gradients, hypoxic and perivascular influences may converge on the same malignant-cell population. Similar plasticity can re-emerge after dissemination, linking CSC-like states in the primary tumor to metastatic adaptation, dormancy and relapse without implying that these states are identical.

### Systemic communication and pre-metastatic niches

The transition from local disease to metastasis begins before tumor cells enter a distant organ. Pre-metastatic niches form when tumor-derived signals and systemic host responses alter vascular permeability, immune composition, fibroblast activity and extracellular matrix at future metastatic sites [5, 23]. Such changes can increase the likelihood that arriving cells are retained and survive, but they do not predetermine metastasis [140]. In experimental models, some distant-tissue changes persist after removal of the primary tumor [141], suggesting that recipient tissues can maintain a conditioned state after the initiating signal has diminished. Although pre-metastatic conditioning is well established experimentally, its timing, anatomical extent and functional importance in patients remain difficult to demonstrate directly.

Extracellular vesicles carry proteins, RNAs, lipids and metabolites between primary tumors and recipient tissues, and their effects depend on the cells that release, transport and receive them. Exosomal integrins have been linked to lung- and liver-directed uptake in experimental models [74, 142–144], but organ selectivity also reflects tumor lineage, vascular access, recipient-cell phenotype and the systemic host response [72, 144, 145]. EVs are one route of pre-metastatic communication rather than an independent niche or a universal code for organotropism.

EV cargo can condition multiple organs. In lung, vesicles alter fibroblast–regulatory T (Treg)-cell interactions [146], activate epithelial TLR3 [72] and deliver hypoxia-regulated RNA [29, 147]; in bone, they influence resident stromal and skeletal cells [148]. Liver-directed vesicles act on Kupffer and stellate cells [149], whereas those reaching brain [150] or lymph nodes [151] can modify barrier and immune function. The recipient tissue—not EV cargo alone—ultimately determines how these signals influence colonization [152].

Platelets provide another systemic link. Tumor-educated platelets undergo molecular changes [153, 154] and support metastasis through immune shielding, adhesion and endothelial interactions [155–159]. Their RNA profiles can report systemic tumor-associated changes [160], although they do not identify the responsible platelet population or mechanism. Platelet-dependent TGF-β signaling [157], vascular recruitment [161] and immune modulation [40] operate alongside EVs and soluble mediators.

### Organ-specific metastatic niches

**Fig. 4:**
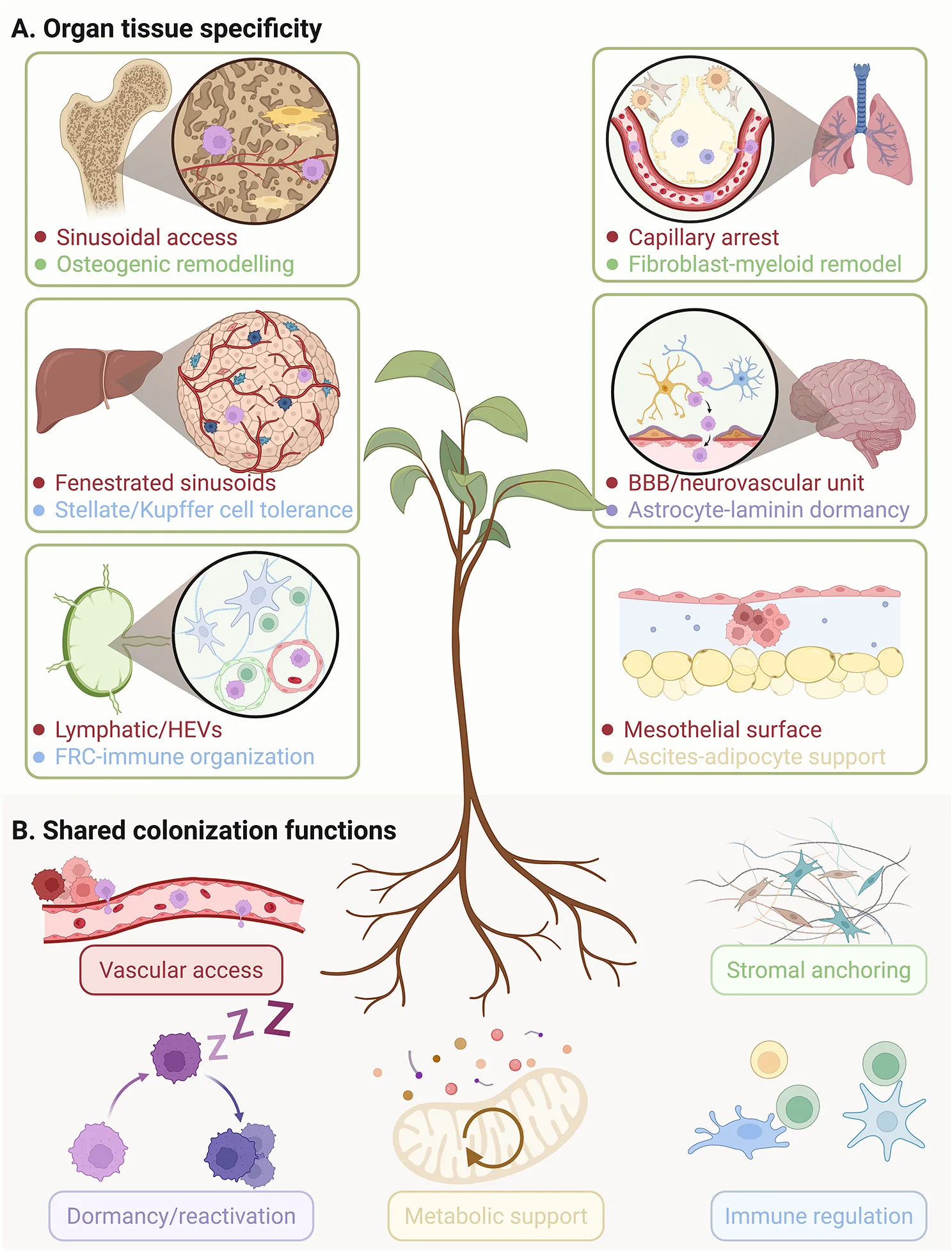
Organ-specific metastatic niches and shared colonization functions. Metastatic niches support shared colonization functions through organ-specific structures and resident-cell populations. (**A**) Organ tissue specificity. Bone and bone marrow, lung, liver, brain, lymph nodes and the peritoneal cavity provide distinct vascular interfaces, stromal cells, immune environments and extracellular matrices that regulate DTC entry, survival and growth. (**B**) Shared colonization functions. Despite their anatomical differences, these niches provide common requirements, including vascular access, stromal anchoring, immune regulation, metabolic support and control of dormancy and reactivation. The branching-tree motif illustrates how shared functional requirements are implemented through organ-specific cellular and structural programs. BBB, blood–brain barrier; DTC, disseminated tumor cell; FRC, fibroblastic reticular cell; HEV, high endothelial venule

Once tumor cells arrive, reciprocal interactions with the recipient tissue establish a metastatic niche. Each organ provides a distinct vascular interface, resident-cell composition, extracellular matrix and metabolic environment. Homing signals such as CXCL12–CXCR4 and VCAM1-mediated adhesion can facilitate colonization, and their disruption reduces metastasis in preclinical models [130]. Despite these anatomical differences, metastatic sites must provide access to tissue, stromal anchoring, immune regulation, metabolic support and control of dormancy or outgrowth (Fig. 4). Evidence is considerably stronger for some organ-specific interactions than for others, and the examples below should not be regarded as equally validated models.

#### Bone metastatic niche

Bone is among the most frequent metastatic sites in advanced breast and prostate cancer [162, 163]. Its sinusoidal vasculature facilitates access to a marrow environment rich in perivascular and endosteal interfaces, although entry alone does not ensure colonization [128, 164].

Stromal anchoring is provided by osteogenic and perivascular cells. Nerve/glial antigen 2 (NG2)-positive bone-marrow stromal cells colocalize with DTCs and support them through N-cadherin-mediated interactions during osteogenic remodeling [39], while heterotypic adherens junctions can activate mTOR signaling in early micrometastases [165]. Tumor-induced changes in osteoblast and osteoclast activity then amplify skeletal growth through the classical vicious cycle [166]. The broader bone microenvironment—including osteoblastic, osteoclastic and vascular components—can be conditioned before tumor-cell arrival and subsequently shapes DTC fate from homing to metastatic outgrowth [28, 167].

The same niche supplies metabolic and immune support. Cancer cells obtain calcium from osteogenic cells through gap junctions [117] and can hijack VCAM1-positive erythroblastic-island macrophages for iron [118]. Osteoblast-derived collagen can suppress NK-cell activity through LAIR1 [168], whereas CCL20–CCR6 signaling between myeloid and T cells contributes to immunosuppression in human prostate-cancer bone metastases and is experimentally targetable [169].

These dependencies create therapeutic opportunities but also expose physiological trade-offs. Chemotherapy-induced Jagged1 in osteoblast-lineage cells can activate Notch signaling and resistance in experimental models [170], and bone metastases may seed additional organs [171]. Interventions must distinguish tumor-co-opted bone functions from those required for normal hematopoiesis and skeletal homeostasis.

#### Lung metastatic niche

The lung combines a dense capillary interface with fibroblast, endothelial and myeloid programs. Breast-cancer metastasis-initiating cells induce an IL-1–CXCL9/10 fibroblast niche that enriches for CXCR3-positive tumor cells [50], while lung mesenchymal stromal cells recruit neutrophils and promote neutrophil extracellular trap (NET) formation through complement-dependent mechanisms [172, 173].

Metabolic and inflammatory states modify this vascular gateway. Endothelial palmitic-acid synthesis activates neutrophil TLR4–NF-κB–LCN2 signaling and weakens vascular integrity in preclinical breast-cancer models [120]. In lung adenocarcinoma precursor lesions, multimodal spatial profiling identified *IL1B*-high macrophage neighborhoods that were more frequent before invasion; experimental IL-1β blockade reduced alveolar progenitor expansion [174].

Endothelial WNT signaling can also influence latency: hypomethylated DTCs with high WNT activity enter quiescence, whereas methylated cells proliferate intravascularly [41]. This mechanism offers a concrete explanation for prolonged intravascular latency in the lung.

#### Liver metastatic niche

The liver combines permissive sinusoidal access with strong local stromal and immune regulation [42, 175, 176]. FAP-positive stellate cells activate NF-κB–NLRP3–caspase-1 signaling and IL-18 secretion [149], while Kupffer cells can initially clear circulating tumor cells but later be reprogrammed to support colonization [177]. Spatial studies show that stellate-cell signals organize Kupffer-cell identity [178], and local lipid exposure supports distinct lipid-associated macrophage phenotypes [175]. Tumor cells must also adapt: transient YAP-controlled plasticity supports early micrometastatic growth before stable CSC hierarchies emerge [179].

#### Brain metastatic niche

The brain metastatic niche is organized around the neurovascular unit, in which endothelial cells, pericytes, astrocytes, microglia and neurons form the blood–brain barrier (BBB) [180]. The intact BBB restricts entry, whereas vascular disruption permits access to the parenchyma [181]. After arrival, granulocyte-derived LCN2 and astrocytic NLRP3–IL-1β signaling can recruit myeloid cells and support growth [182, 183]. Astrocyte-derived laminin-211 provides a growth-restraining perivascular dormancy cue, discussed below, and microglia can either constrain or support colonization depending on state and context [184, 185]. This combination of restricted vascular access and specialized perivascular regulation distinguishes the brain from sinusoidal organs.

#### Lymph node metastatic niche

The lymph node functions as an immune and stromal relay rather than a passive staging site. Before tumor-cell arrival, lymphangiogenesis, ECM remodeling and recruitment of immunosuppressive cells can alter the nodal environment [186, 187]. Fibroblast-derived periostin promotes lymphatic endothelial function [188], ITGA6-containing EVs engage CD151 on lymphatic endothelial cells [150], and sentinel-node vasculature can be reorganized [189]. After colonization, lymphatic spread may contribute to further dissemination [190]. Spatial profiling of human HNSCC and a complementary mouse model identified myeloid–CAF interfaces that suppress T-cell function and activate Tregs [187]; related signatures in adjacent tumor-free nodes suggest that regional immune tolerance can extend beyond the involved node [187].

#### Peritoneal metastatic niche

The peritoneal cavity is a distinctive metastatic site for ovarian and gastrointestinal cancers, with ascites acting as a dynamic niche component [191]. Ascites weakens the mesothelium, promotes integrin-dependent implantation of tumor spheroids [192], and imposes mechanical compression that favors metastatic success [193]. Mesothelial cells support spheroid formation [192] and undergo mesothelial-to-mesenchymal transition in response to tumor-derived signals [194]. TROP2-enriched EVs can amplify this transition through TNF-α–NF-κB signaling [151]. Single-cell analyses further implicate ascites in remodeling primary and metastatic ovarian-cancer ecosystems [195], establishing peritoneal fluid as an active signaling and mechanical environment rather than a passive medium.

#### Malignant bone-marrow niches: a non-metastatic comparison

The bone marrow provides a non-metastatic comparison because hematological malignancies remodel the stromal infrastructure of hematopoiesis. In multiple myeloma, stromal cells promote T-cell suppression [196], epigenetic reprogramming [197] and drug resistance [198]. Osteocyte-derived EVs stabilize HIF-1α and promote CSC-like chemoresistance [199], whereas stromal c-FOS signaling induces ABCG2 and aldehyde dehydrogenase [200]. Single-cell [201] and spatial studies [202] reveal patient-specific immune and subclonal organization. Leukemic stem cells likewise exploit retention and survival signals, including CXCL12–CXCR4 [203]. These findings underscore the need to disrupt malignant niche dependence without damaging hematopoietic stem-cell maintenance.

#### Shared principles across organ-specific metastatic niches

Comparison across organs reveals common biological requirements but different anatomical solutions. Sinusoidal vessels facilitate access to bone marrow and liver, whereas the blood–brain barrier restricts entry into the brain [42, 106, 180, 204]. Osteogenic cells anchor tumor cells in bone, fibroblasts remodel lung tissue, stellate cells organize hepatic niches and astrocytes shape the brain perivascular environment [42, 49, 165, 185]. These resident populations do not act alone: their effects depend on local vascular, immune, metabolic and mechanical conditions.

This local context helps explain organ selectivity. Inflammatory or suppressive immune states impose different constraints on disseminated-cell survival, while calcium availability in bone, organ-specific lipid metabolism in the lung and liver, and adipocyte-derived resources in the peritoneal cavity provide different forms of metabolic support [4, 117, 193]. Dormancy signals are likewise tissue-dependent and remain incompletely resolved in several organs [38, 139]. Organotropism emerges when a tumor-cell state is compatible with the way a particular tissue supplies these shared functions; neither the tumor nor the organ determines the pattern alone.

### Dormancy and reactivation niches

**Fig. 5:**
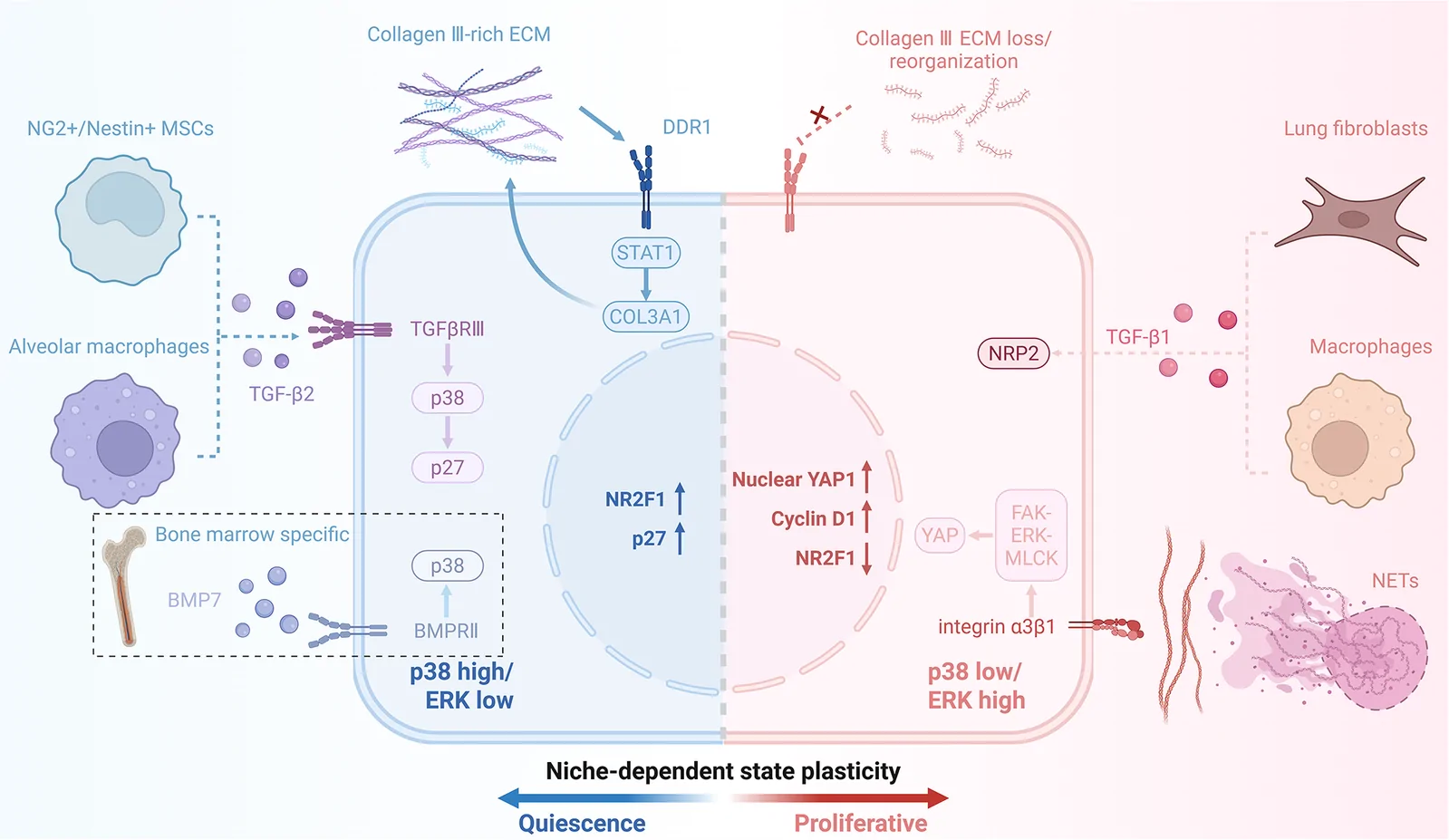
Niche-regulated pathways governing disseminated tumor-cell dormancy and reactivation. DTC fate is shaped by vascular, matrix, stromal and inflammatory signals that favor dormancy (blue) or reactivation (red). Dormancy-promoting inputs—including stable microvascular TSP-1, collagen III–DDR1–STAT1 signaling and TGF-β2/BMP pathways—converge on p38-high/ERK-low activity, NR2F1 and p27 induction, and cell-cycle arrest. Matrix remodeling, sprouting-vessel signals, TGF-β1–NRP2 signaling and inflammation-associated laminin cleavage activate integrin–FAK–ERK–MLCK and YAP pathways that favor p38-low/ERK-high activity, cyclin D1 expression and metastatic outgrowth. The mechanisms shown derive from complementary organ and experimental contexts and need not operate simultaneously. BMPRII, bone morphogenetic protein receptor II; DTC, disseminated tumor cell; FAK, focal adhesion kinase; MLCK, myosin light-chain kinase; MSC, mesenchymal stromal cell; NE, neutrophil elastase; NET, neutrophil extracellular trap; NRP2, neuropilin 2; POSTN, periostin; TGFβRIII, transforming growth factor-β receptor III; TSP-1, thrombospondin 1

For DTCs that survive initial seeding, dormancy provides an alternative to immediate outgrowth and is a major source of late metastatic relapse [205]. Dormancy may occur at the level of individual cells, through failure of angiogenic expansion or through immune-mediated equilibrium [205, 206]. In cellular dormancy, a high p38-to-ERK activity ratio promotes arrest mediated by nuclear receptor subfamily 2 group F member 1 (NR2F1) and p27, whereas ERK activity favors cyclin D1-dependent proliferation [206] (Fig. 5). Dormant DTCs and metastasis-initiating cells can share progenitor-like programs that confer stress tolerance, plasticity and immune evasion [207, 208], and can use organ-specific signals such as periostin and tenascin C to reinforce WNT and Notch activity [208]. Dormancy, stemness and treatment resistance intersect, but remain biologically distinct states.

Dormancy is sustained by tumor-generated matrix and organ-derived signals. Type III collagen maintains DDR1–STAT1 and p38-high/ERK-low signaling [26]. TGF-β2 and BMP inputs induce quiescence in bone marrow and lung [209, 210]. Additional organ-specific signals include astrocytic laminin-211 in brain [184], vascular and endosteal shelter in bone [128], and endothelial WNT [41] and PEAR1 [211] in lung.

Reactivation follows loss of quiescence-maintaining relationships. Lung-derived TGF-β1–NRP2 signaling promotes DTC outgrowth [212]. During inflammation, NET-associated proteases expose a laminin epitope that activates integrin–FAK–ERK–MLCK signaling and YAP [25]. This creates a therapeutic dilemma: dormancy might be stabilized through circuits such as collagen III–DDR1–STAT1 [26], or dormant cells awakened and treated. Either approach requires identifying and eliminating the relevant cells without accelerating otherwise indolent disease.

Dormancy should not be inferred from slow proliferation or a static molecular signature alone. Its defining evidence is longitudinal: a reversible period of growth arrest followed by reactivation. This standard matters clinically because the choice among maintaining quiescence, inducing awakening and directly eliminating dormant cells depends on knowing that a true dormant state exists and can be monitored over time. More broadly, the same need to distinguish association from changing cell fate applies to every cancer niche and motivates the methodological approaches considered next.

## Emerging technologies for niche discovery

Cataloging tumor components is only the first step toward identifying a cancer niche. Candidate interactions must be placed within tissue architecture, followed over time and tested for their effects on malignant-cell behavior. Spatial, longitudinal and perturbational approaches provide complementary evidence for interpreting niche-mediated progression and treatment resistance.

### Mapping niche composition and spatial organization

**Fig. 6:**
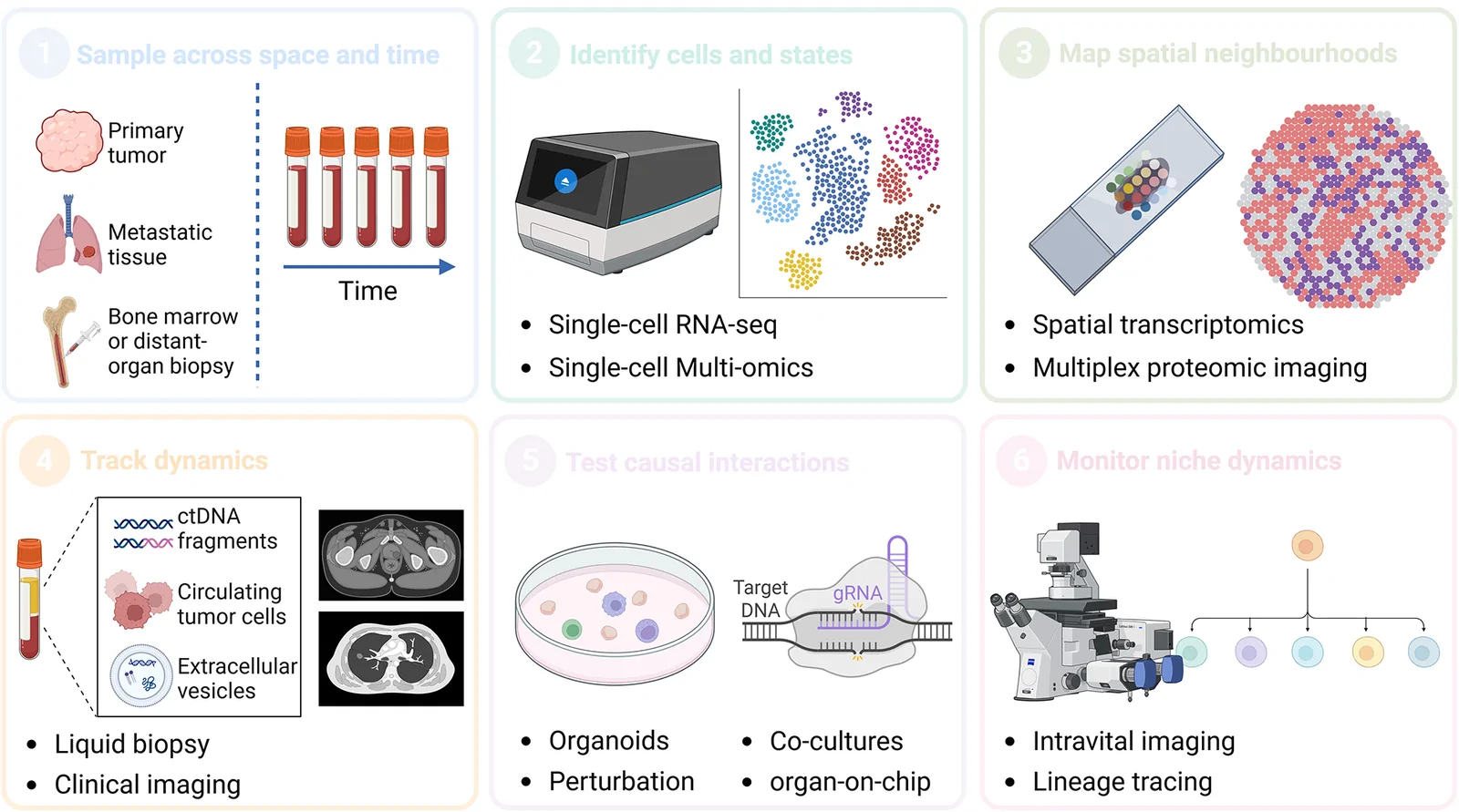
An integrated workflow for identifying, validating and longitudinally characterizing cancer niches. (1) Spatially and temporally resolved samples are collected from primary tumors, metastatic tissues, bone marrow or distant-organ biopsies, including serial specimens obtained during disease progression and treatment. (2) Single-cell RNA sequencing and single-cell multi-omics define the cellular composition, molecular states and candidate interactions within each niche. (3) Spatial transcriptomics and multiplex protein imaging localize these cells and states within intact tissue and resolve their neighborhood organization. (4) Liquid-biopsy analytes—including circulating tumor DNA (ctDNA), circulating tumor cells and extracellular vesicles—together with clinical imaging enable minimally invasive tracking of niche-associated dynamics. (5) Organoids, co-culture and organ-on-chip systems, combined with targeted perturbation, test how candidate cells, signals and interactions influence tumor behavior. (6) Intravital imaging and lineage tracing monitor cell fate and niche evolution over time. Together, these complementary approaches connect molecular profiling and spatial association with mechanistic validation and longitudinal assessment, showing which niche features are reproducible, functionally important and relevant to patient care

Single-cell sequencing resolves cellular states and candidate signaling, while spatial methods place them within tissue architecture [164, 213, 214]. In bone marrow, integration with co-detection by indexing (CODEX) imaging identified organized mesenchymal stromal cell (MSC) subsets and altered blast–MSC neighborhoods in acute myeloid leukemia (AML) [164]. A pan-cancer analysis similarly defined 39 non-immune stromal states linked to spatial and clinical features [30]. Other studies have mapped suppressive myeloid–stromal neighborhoods in prostate cancer [215], *IL1B*-high macrophage niches in lung precursor lesions [174] and tumor-promoting Treg states in clear-cell renal cell carcinoma (RCC) [216]. Such atlases reveal composition, proximity and candidate communication, but not whether a neighborhood controls malignant-cell behavior (Fig. 6).

### Observing niche evolution

Intravital imaging adds a temporal dimension to spatial observation, allowing individual cells and local tissue changes to be followed in living models. Serial imaging of breast-cancer DTCs in the brain showed that escape from a single-cell or micrometastatic state is a rate-limiting step in colonization [184]. Second-harmonic generation two-photon microscopy has likewise visualized changes in type III collagen architecture during the transition from dormancy to reactivation [26]. These approaches can connect a changing niche feature with subsequent cell behavior, although their field of view, duration and applicability to human disease remain limited.

In patients, repeated tissue sampling is often impractical, making liquid biopsy and clinical imaging important. Circulating tumor cells, circulating tumor DNA (ctDNA) and circulating EVs can report on tumor burden and metastatic processes [217], with multiparametric monitoring explored in metastatic breast cancer [218], NSCLC [219] and gastrointestinal cancers [220]. Broader circulating-biomarker evidence has also been assessed in breast cancer [221] and glioblastoma [222]. However, these approaches have limited anatomical and functional resolution: a circulating signal rarely identifies its tissue source or establishes that it drives a proposed niche interaction [220].

### Perturbing niche interactions

Organoids and co-culture systems permit controlled manipulation of candidate interactions. Colorectal-cancer organoids recapitulate YAP-dependent plasticity during liver metastasis [179], and multiregional models reproduce fibroblast-induced oncofetal states [133]. Organ-on-chip systems model systemic processes, including lung pre-metastatic conditioning [223], chimeric antigen receptor T-cell (CAR-T-cell) interactions in bone marrow [224] and ovarian-cancer colonization of vascularized omentum [225]. Additional three-dimensional models reproduce myeloma–stromal crosstalk [226], while microfluidic systems resolve trafficking through marrow sinusoids [204]. These platforms provide experimental control but require confirmation in intact tissues or patients.

No single method captures all of these features. Spatial maps identify candidate interactions, longitudinal approaches establish their timing and perturbation studies test their contribution to tumor behavior. Reproduction across models and association with measurable features in patient samples can then support biomarker and treatment development. These standards are especially important when studying therapy resistance, because treatment may create or reshape the very niche that protects residual disease.

## Niche-mediated therapy resistance

Therapy resistance emerges from interactions between malignant cells and treatment-altered tissue. Changes in vascular function, matrix organization, metabolism and immune composition can protect residual cells, favor stem-like or dormant phenotypes and support repopulation. Across treatment modalities, physical barriers limit drug or immune-cell access, stromal signals suppress cell death, hypoxic and metabolic adaptation reduce sensitivity and local immune conditions blunt antitumor responses. Niche-directed therapy seeks to interrupt the interactions on which resistant cells depend, rather than simply remove a stromal cell type (Tables 2 and 3).

### Physical barriers and stromal survival signals

Physical exclusion and paracrine survival are closely linked. Dense or poorly organized matrix can restrict drug penetration and immune-cell movement [31], while CAF-derived factors, metabolites and EV cargo alter cell-death pathways and immune function [47]. Stromal support can also accelerate repopulation between treatment cycles [227]. These diverse EV-mediated effects remain difficult to target as a single mechanism [47].

LOX-mediated collagen cross-linking illustrates how a defined matrix process can be linked to treatment response. Pan-LOX inhibition with PXS-5505 reduces desmoplasia and improves gemcitabine response in pancreatic-cancer models [228], while LOX targeting increases drug penetration and reduces chemotherapy resistance in models of triple-negative breast cancer [229]. These studies connect matrix remodeling to drug delivery and response, although clinical benefit has not yet been established.

Resistance can emerge from multicellular stromal and immune networks. Myeloid, lymphoid, mesenchymal, adipocyte and endothelial populations have been linked to immune suppression, EMT, drug efflux, DNA repair and tissue remodeling [230–234]. Because these functions overlap and also support normal tissue homeostasis, selective disruption of a resistance-producing interaction is more plausible than depletion of an entire stromal population.

Radiotherapy shows how treatment can generate the niche that subsequently limits response. Fibroblast activation protein (FAP)-positive CAFs, suppressive myeloid cells, extracellular matrix and abnormal vasculature can reduce radiosensitivity [235]. Radiation-recruited monocytes may differentiate into M2-like tumor-associated macrophages (TAMs) that release TGF-β and IL-10, suppress immunity and reinforce fibroblast activation [236]. CAF depletion, CSF1R-dependent macrophage reprogramming and matrix modification are candidate radiosensitizing approaches, but their effects are likely to depend on tumor type, dose and treatment sequence.

### Hypoxic adaptation and therapeutic resistance

Hypoxia confers resistance both directly and through cellular adaptation. Limited oxygen reduces radical formation during radiotherapy, while HIF-dependent programs alter metabolism, drug efflux and DNA repair [16, 17]. HIF-2α can also reinforce OCT4- and SOX2-associated stem-like states [16], linking oxygen limitation to plasticity and persistence. Selective HIF-2α inhibition is clinically active in genetically defined clear-cell renal cell carcinoma, but this does not establish that combined HIF-1α/HIF-2α inhibition or hypoxia-directed treatment will be effective in unselected tumors [17, 111].

Belzutifan provides a specific clinical precedent. In LITESPARK-004, it produced durable responses in VHL-associated RCC [237]; related studies confirmed activity, with anemia and hypoxia as prominent toxicities [238]. Its approval supports HIF-2α inhibition in VHL-deficient tumors, not hypoxic niches as a general treatment class [238].

Hypoxia-activated prodrugs test a different premise: selective drug release within oxygen-poor tissue. Evofosfamide releases a DNA cross-linker under hypoxia [239], but failed to improve overall survival when added to doxorubicin in phase III soft-tissue sarcoma [240]. A phase I combination with ipilimumab showed manageable safety and some responses [241]. The contrast with belzutifan shows why a plausible niche mechanism is insufficient without prospective measurement of the relevant state and confirmation that treatment altered it.

### Immune suppression and organ-specific resistance

Immune resistance is similarly shaped by local and systemic context. Cellular crosstalk, EV signaling and metabolic competition can suppress T-cell priming, trafficking and effector function [242, 243], while individual metastatic sites may contribute differently to systemic immune escape [244]. In colorectal cancer, liver metastases are relatively tolerogenic and myeloid-rich, whereas lung metastases are more inflammatory [245]. Metastatic site should be considered in mechanistic stratification rather than treated only as a staging variable.

Vascular–immune combinations demonstrate the distinction between clinical efficacy and niche-guided treatment. IMbrave150 improved overall survival with atezolizumab plus bevacizumab in hepatocellular carcinoma [33]. CheckMate 9ER improved multiple efficacy outcomes with nivolumab plus cabozantinib in advanced RCC [34]. Neither trial selected patients by vascular or immune niche state, and any contribution from vascular normalization remains inferential.

The same boundary applies to tumor-intrinsic biomarkers. Pembrolizumab received tissue-agnostic approval for MSI-H or mismatch repair-deficient solid tumors [246]. Mismatch-repair deficiency shapes the immune environment but does not directly measure a cancer niche.

Resistance is co-produced by tumor evolution and treatment-induced niche remodeling. A therapy may remove sensitive cells while leaving structural protection, metabolic support or immune suppression intact, and may itself induce new protective interactions. Sequence and timing become part of the mechanism when vascular normalization, matrix remodeling or immune reprogramming is intended to create a transient window for another treatment.

## Translating niche-directed therapy

Translating niche biology raises three questions: whether an interaction operates and can be measured in human disease; whether it can be disrupted without unacceptable damage to normal tissue; and when intervention is most likely to improve outcome. Several therapies already alter components implicated in cancer niches, yet none has used direct measurement of a niche dependency to select patients. Niche-guided treatment selection remains unestablished.

The first question is whether a proposed dependency operates in human tissue. Preclinical studies implicate matrix cross-linking [228, 229], FAP-positive stromal programs [247], organ-specific metabolic exchange [117, 119], suppressive myeloid and fibroblast circuits [49, 168, 248], dormancy and reactivation signals [26, 212], and altered oxygen delivery [249]. Translation requires evidence that these interactions persist in human disease and contribute to progression or resistance.

Neural and TGF-β-directed approaches expose this gap. β-adrenergic blockade has perioperative biomarker data but no validated method for identifying tumors driven by neural signaling [250], whereas NGF–TRK and perineural-invasion-directed approaches remain investigational. Galunisertib combinations have shown tolerability and modest activity in pancreatic cancer without establishing a niche-specific clinical effect [251, 252]. In hematological malignancies, CAR-T therapies demonstrate potent tumor-directed activity, but marrow immune suppression, metabolic competition and stromal barriers can limit persistence [253–255]. Here, niche modulation is more plausibly used to support an established cellular therapy than as an independently validated treatment class.

The first question also requires evidence that an intervention changes the intended tissue interaction. Spatial profiling, imaging, immune phenotyping and liquid biopsy may contribute, but few assays have standardized thresholds or prospective evidence for treatment selection. TLS assessment remains promising but unstandardized [58, 65], while evofosfamide illustrates the risk of testing a hypoxia-directed drug without measuring the relevant state [239]. Comparable measurements are needed for matrix, neural and metabolic dependencies.

The second question is whether the interaction can be disrupted without unacceptable damage to normal tissue. NG2-positive cells participate in both bone remodeling and metastatic colonization [39]; erythroblastic-island macrophages support erythropoiesis but can be co-opted by tumors [118]; and Kupffer cells maintain hepatic immune surveillance yet can be reprogrammed during metastasis [177]. Intervention must distinguish a tumor-induced function from the normal role of the same cell population—for example, by inhibiting pathological osteoclast activation without broadly suppressing bone remodeling.

The third question concerns disease setting and timing. Persistent distant-tissue reprogramming after primary-tumor removal in mouse models [141] raises the possibility of adjuvant niche-directed treatment. The aim would not be shrinkage of an established lesion, but rather prevention of pre-metastatic conditioning, durable suppression or elimination of residual disseminated cells, and avoidance of later reactivation. Testing this hypothesis will require trial designs and endpoints suited to recurrence risk and time to metastatic outgrowth.

Timing also matters when niche modulation is combined with established therapy. Radiotherapy can generate macrophage-rich radioprotective phenotypes [236], while chemotherapy can activate CAF–EV resistance programs [47]. Vascular normalization or matrix remodeling may create only transient opportunities for immune or cytotoxic therapy, making sequence integral to efficacy. Repeated tissue or circulating measurements could guide treatment adaptation, but this approach has not yet been prospectively validated [256].

A practical translational sequence begins by testing candidate dependencies in intact models or ex vivo human tissues rather than inferring them from expression or spatial association alone. Early trials should then confirm that the intended tissue interaction changed and use endpoints suited to the proposed effect. Delayed recurrence, prolonged dormancy or improved response to a partner therapy may be more informative than short-term tumor shrinkage.

**Table 2:** Preclinical niche-targeted therapies

Target	Agent/strategy	Mechanism	Cancer type	Model	Key findings	Ref
Calcium signaling	As₂O₃, danusertib	Blocks calcium influx from osteogenic cells	Bone metastasis (breast)	Mouse	Reduces bone metastatic colonization	[117]
HAO1	HAO1 inhibitors	Suppresses oxalate metabolism/NET formation	Lung PMN	Mouse	Reduces lung PMN formation	[119]
LOX family	Pan-LOX inhibitor (PXS-5505)	Blocks collagen cross-linking/ECM stiffening	Pancreatic cancer	Mouse	Impairs stromal remodeling, enhances gemcitabine response	[228]
LOX	LOX inhibitors	Reduces collagen cross-linking, increases drug penetration	TNBC	Mouse/in vitro	Overcomes chemotherapy resistance	[229]
FAPα	FAPα-activated prodrugs	Disrupts liver PMN	Liver metastasis	Mouse	Ameliorates PMN formation	[247]
SCUBE2	Anti-SCUBE2 antibody	Disrupts osteoblastic niche immune suppression	Bone metastasis (breast)	Mouse	Suppresses bone metastasis	[168]
MMP-13	MMP-13 targeting	Reverses postoperative immunosuppression	Multiple	Mouse	Mitigates persistent immunosuppression	[141]
IL-12 delivery	GEMys (IL-12)	Rebalances immune suppression in PMN	Multiple	Mouse	Rebalances core immune suppression	[248]
PGE2	Fibroblast-specific Ptgs2 deletion	Blocks CAF-mediated immune suppression	Lung metastasis	Mouse	Improves DC therapy + PD-1 blockade (genetic proof-of-concept)	[49]
Type III collagen	Collagen enrichment	Induces/maintains dormancy	Multiple	Mouse	Sustains DTC dormancy via DDR1/STAT1	[26]
TGFβ1-NRP2	NRP2 axis modulation	Prevents dormancy escape	Lung metastasis	Mouse	Blocks reactivation from dormancy	[212]
β-adrenergic signaling	Propranolol	Blocks ADRB2-mediated tumor growth	Gastric, colorectal	Mouse	Reduces tumor growth, enhances anti-CTLA-4 immunotherapy	[90, 92]
Exosomal integrins	Integrin-blocking antibodies	Redirects EV trafficking	Breast	Mouse	Reduces organ-specific metastasis	[74]
Jagged1/Notch	Anti-Jagged1 antibody (15D11)	Blocks Jagged1-Notch signaling in osteoblastic niche; reverses chemotherapy-induced niche resistance	Bone metastasis (breast)	Mouse	Synergizes with paclitaxel to suppress bone metastasis	[170]
Endothelial fatty acid synthesis	GLP-1R agonists	Suppresses endothelial palmitic acid production; prevents neutrophil LCN2 secretion and vascular barrier disruption	Lung metastasis (breast)	Mouse	Preserves vascular integrity, suppresses lung metastasis	[120]
CCL20/CCR6	CCL20-CCR6 axis inhibitors	Disrupts myeloid CCL20–T cell CCR6 immunosuppressive signaling in bone metastasis	Bone metastasis (prostate)	Mouse/Human scRNA-seq	Restores T-cell reactivity, prolongs survival	[169]

CAF, cancer-associated fibroblast; DTC, disseminated tumor cell; ECM, extracellular matrix; EV, extracellular vesicle; FAP, fibroblast activation protein; GEMy, genetically engineered myeloid cell; HAO1, hydroxyacid oxidase 1; LOX, lysyl oxidase; MM, multiple myeloma; NET, neutrophil extracellular trap; NRP2, neuropilin-2; PMN, pre-metastatic niche; TNBC, triple-negative breast cancer. Agents that have also entered clinical investigation are included when preclinical studies provide direct evidence of a niche-mediated mechanism.

**Table 3:** Selected clinical and translational studies relevant to niche targeting

Target	Agent	Trial/Phase	Cancer type	Status	Key results	Ref
HIF-2α	Belzutifan (MK-6482)	Phase II (LITESPARK-004)	VHL-associated RCC	FDA-approved (2021)	ORR 49% (initial) → 67% (50-mo follow-up); durable responses	[237, 257]
RANKL	Denosumab	Phase III	Nonmetastatic castration-resistant prostate cancer	Completed; preventive indication not approved	Improved bone-metastasis-free survival; no overall-survival benefit	[258]
RANKL/osteoclast axis	Denosumab vs. zoledronic acid	Phase III	Newly diagnosed MM	Completed; both agents clinically used	Denosumab noninferior for first SRE (HR 0.98)	[259]
VEGF + PD-L1	Bevacizumab + atezolizumab	Phase III (IMbrave150)	HCC	FDA-approved	Median OS 19.2 vs. 13.4 mo vs. sorafenib (HR 0.66)	[33]
VEGFR + PD-1	Cabozantinib + nivolumab	Phase III (CheckMate 9ER)	Advanced RCC	FDA-approved	Median PFS 16.4 vs. 8.3 mo (HR 0.58); OS 46.5 vs. 35.5 mo (HR 0.79)	[34, 260]
PD-1 + CTLA-4	Nivolumab + ipilimumab	Translational study (human cohorts + mouse models)	NSCLC brain metastases	Published	Clinical analyses suggested improved intracranial control; TLS-like structures shown in mice	[69]
β-adrenergic	Propranolol	Phase II (randomized)	Breast cancer (perioperative)	Completed	Downregulated metastasis biomarkers (Snail/Slug, NF-κB); increased immune infiltration	[250]
TGF-β RI	Galunisertib + gemcitabine	Phase II	Pancreatic cancer	Completed	Median OS 8.9 vs. 7.1 mo (HR 0.79)	[251]
TGF-β RI + PD-L1	Galunisertib + durvalumab	Phase Ib/II	Metastatic pancreatic cancer	Completed	Tolerable but limited clinical activity	[252]
Hypoxia-activated prodrug	Evofosfamide (TH-302) + doxorubicin	Phase III (TH CR-406/SARC021)	Advanced soft-tissue sarcoma	Completed	Median OS 18.4 vs. 19.0 mo (HR 1.06, *P* = 0.527); did not meet primary endpoint	[240]
PD-1	Pembrolizumab	Phase II (KEYNOTE-158)	MSI-H/dMMR solid tumors (tissue-agnostic)	FDA-approved	ORR 34.3% across noncolorectal MSI-H cancers	[246]
BCMA CAR-T	Idecabtagene vicleucel (ide-cel)	Phase III (KarMMa-3)	Relapsed/refractory MM	FDA-approved	Median PFS 13.3 vs. 4.4 mo (HR 0.49)	[253]
BCMA CAR-T	Ciltacabtagene autoleucel (cilta-cel)	Phase II (CARTITUDE-1)	Relapsed/refractory MM	FDA-approved	ORR 98%	[254]
CXCR4	Plerixafor	Phase I (plerixafor + rituximab)	Chronic lymphocytic leukemia	Completed	Feasible CXCR4-mediated niche disruption; efficacy not established	[261]
TLR agonist-based vaccination	TLR agonists + dendritic-cell vaccination	Melanoma vaccine studies; randomized phase II glioma trial	Melanoma; malignant glioma	Published	Immune-site and interferon responses characterized; clinical TLS induction not established	[262, 263]

HCC, hepatocellular carcinoma; HIF, hypoxia-inducible factor; ICI, immune checkpoint inhibitor; MM, multiple myeloma; MSI-H, microsatellite instability-high; dMMR, mismatch repair-deficient; ORR, objective response rate; OS, overall survival; PFS, progression-free survival; RCC, renal cell carcinoma; SRE, skeletal-related event; TLS, tertiary lymphoid structure; VEGF, vascular endothelial growth factor; VHL, von Hippel-Lindau.

## Future perspectives

An unresolved question is which niche changes are transient and which persist long enough to influence progression or relapse. Wound-repair-like fibroblast and matrix programs can create permissive fields before overt malignancy [264], and trophocyte-like CAFs can induce oncofetal plasticity early in colorectal cancer [133]. Neither observation establishes a universal sequence from tissue injury to cancer. Longitudinal studies must determine when these programs arise, whether they regress after removal of the initiating stimulus and which retain biological importance during treatment or dissemination.

Systemic communication is difficult to resolve in patients. Primary tumor, bone marrow, circulation and recipient organs may form relay networks; the HTRA1–MMP-13 marrow relay provides experimental proof of principle [141]. Other studies implicate bone-metastatic stromal programs [168] and immune circuits [169], platelet RNA changes [161] and treatment-induced CAF remodeling [235]. Their relevance will depend on tumor subtype, organ and treatment history. Paired longitudinal sampling across tumors, blood and metastatic tissues is needed to identify reproducible networks.

Therapeutic selectivity remains a further challenge. Specific dependencies—such as metabolic exchange, matrix-mediated drug exclusion, dormancy signals or suppressive immune circuits—are more tractable than broadly expressed niche markers. Spatial profiling can identify candidate phenotypes [174, 265, 266], longitudinal imaging can reveal when they matter [26, 184], and organoid or microphysiological models can test intervention [224, 225, 267]. Translation requires repeatable patient measurements and evidence that altering the interaction improves a meaningful outcome. Computational spatial pathology may eventually convert tissue patterns into practical classifiers, but will require external validation and a clear biological basis.

## Conclusion and outlook

Cancer niches are dynamic relationships through which local tissues shape malignant-cell behavior and are, in turn, remodeled during progression and treatment. Vascular, stromal, immune and neural interactions generate hypoxic, metabolic and mechanical conditions that support stemness, dissemination, dormancy or outgrowth. By organizing these relationships according to tissue structure, local condition and disease state, this framework moves beyond inventories of tumor-microenvironment components toward the interactions that control malignant-cell fate.

These relationships extend beyond the primary tumor. Circulating signals and host responses condition distant tissues before tumor-cell arrival, after which organ-specific environments determine whether disseminated cells are eliminated, remain dormant or grow. Different organs meet the requirements for colonization through distinct resident cells and tissue architectures, helping to explain organ-selective metastasis without reducing it to a single tumor signal or tissue property.

Treatment introduces another stage of niche remodeling: it removes sensitive cells but can leave or induce local conditions that protect residual disease. Translating niche biology will require evidence that a specific interaction contributes to progression or resistance, that treatment alters it in the relevant tissue and that the patients most likely to benefit can be identified. With greater spatial and temporal resolution, cancer-niche research may guide more rational treatment combinations and open new opportunities to prevent relapse and metastatic outgrowth.

## Data Availability

No datasets were generated or analysed during the current study.

## Abbreviations

ADRB2: β2-adrenergic receptor
AML: Acute myeloid leukemia
BBB: Blood-brain barrier
BDNF: Brain-derived neurotrophic factor
CAF: Cancer-associated fibroblast
CAR-T: Chimeric antigen receptor T cell
CHRM1: Cholinergic receptor muscarinic 1
CHRM3: Cholinergic receptor muscarinic 3
CODEX: Co-detection by indexing
CSC: Cancer stem cell
CTC: Circulating tumor cell
ctDNA: Circulating tumor DNA
CTLA-4: Cytotoxic T-lymphocyte-associated protein 4
DDR1: Discoidin domain receptor 1
dMMR: Mismatch repair-deficient
DTC: Disseminated tumor cell
ECM: Extracellular matrix
EMT: Epithelial–mesenchymal transition
EV: Extracellular vesicle
FAP: Fibroblast activation protein
G-CSF: Granulocyte colony-stimulating factor
GDNF: Glial cell line-derived neurotrophic factor
GEMy: Genetically engineered myeloid cell
GLP-1: Glucagon-like peptide-1
GMP: Granulocyte-monocyte progenitor
HAO1: Hydroxyacid oxidase 1
HEV: High endothelial venule
HIF: Hypoxia-inducible factor
HNSCC: Head and neck squamous cell carcinoma
HR: Hazard ratio
ICI: Immune checkpoint inhibitor
LCN2: Lipocalin-2
LOX: Lysyl oxidase
LPA: Lysophosphatidic acid
LSC: Leukemic stem cell
MDSC: Myeloid-derived suppressor cell
MM: Multiple myeloma
MSC: Mesenchymal stromal cell
MSI-H: Microsatellite instability-high
mTOR: mechanistic target of rapamycin
NET: Neutrophil extracellular trap
NGF: Nerve growth factor
NK: Natural killer
NRP2: Neuropilin-2
NSCLC: Non-small cell lung cancer
ORR: Objective response rate
OS: Overall survival
PD-1: Programmed cell death protein 1
PD-L1: Programmed death-ligand 1
PFS: Progression-free survival
PGE2: Prostaglandin E2
PKD: Protein kinase D
PMN: Pre-metastatic niche
PNI: Perineural invasion
RCC: Renal cell carcinoma
scRNA-seq: Single-cell RNA sequencing
SRE: Skeletal-related event
TACR1: Tachykinin receptor 1
TAM: Tumor-associated macrophage
TLR7: Toll-like receptor 7
TLS: Tertiary lymphoid structure
TNBC: Triple-negative breast cancer
Treg: Regulatory T cell
VEGF: Vascular endothelial growth factor
VHL: Von Hippel–Lindau
